# An Efficient Virtual Machine Consolidation Scheme for Multimedia Cloud Computing

**DOI:** 10.3390/s16020246

**Published:** 2016-02-18

**Authors:** Guangjie Han, Wenhui Que, Gangyong Jia, Lei Shu

**Affiliations:** 1 Department of Information and Communication Systems, Hohai University, 200 North Jinling Road, Changzhou 213022, China; jumperquejust@gmail.com; 2 Department of Computer Science, Hangzhou Dianzi University, Hangzhou 310018, China; gangyong@hdu.edu.cn; 3 Guangdong Petrochemical Equipment Fault Diagnosis Key Laboratory, Guangdong University of Petrochemical Technology, Guangdong 525000, China; lei.shu@ieee.org

**Keywords:** multimedia cloud computing, virtual machine (VM) migration, energy consumption, QoS

## Abstract

Cloud computing has innovated the IT industry in recent years, as it can delivery subscription-based services to users in the pay-as-you-go model. Meanwhile, multimedia cloud computing is emerging based on cloud computing to provide a variety of media services on the Internet. However, with the growing popularity of multimedia cloud computing, its large energy consumption cannot only contribute to greenhouse gas emissions, but also result in the rising of cloud users’ costs. Therefore, the multimedia cloud providers should try to minimize its energy consumption as much as possible while satisfying the consumers’ resource requirements and guaranteeing quality of service (QoS). In this paper, we have proposed a remaining utilization-aware (RUA) algorithm for virtual machine (VM) placement, and a power-aware algorithm (PA) is proposed to find proper hosts to shut down for energy saving. These two algorithms have been combined and applied to cloud data centers for completing the process of VM consolidation. Simulation results have shown that there exists a trade-off between the cloud data center’s energy consumption and service-level agreement (SLA) violations. Besides, the RUA algorithm is able to deal with variable workload to prevent hosts from overloading after VM placement and to reduce the SLA violations dramatically.

## 1. Introduction

The cloud computing paradigm [1] has rapidly attracted much of the public’s attention in recent years, as it can delivery subscription-based services to users in the pay-as-you-go model. It is convenient for users who come from different places in the world to access the cloud services easily, which are usually described as infrastructure as a service (IaaS), platform as a service (PaaS) and software as a service (SaaS). Besides, these cloud services also benefit these developers who have innovative ideas for new Internet services, since they are no longer required to make large capital outlays in the hardware and software infrastructures to deploy their services or human expense to implement their ideas [2].

With the rapid development of cloud computing, multimedia cloud computing is emerging to provide a variety of media services on the Internet. There exists millions of multimedia services and applications over the Internet every day, and the cloud data center can provide a significant amount of storage and computation to support multimedia applications. Therefore, multimedia users can install their multimedia applications on the cloud easily and process them in a distributed manner. However, how to reduce the large energy consumption caused by multimedia cloud computing while satisfying the multimedia applications’ quality of service (QoS) is still a challenge for researchers. Since the base of multimedia cloud computing is the infrastructures provided by cloud data centers, the problem can be described as reducing energy consumption and improving system performance in the cloud data center.

The cloud data center is usually made up of a large number of well-configured and interconnected physical facilities [3]. With the growth in the number of large data centers, the energy consumption of these hardware facilities has become a general public concern. Statistics show that the average energy consumption of a data center is equivalent to that consumed by 25,000 households [4]. Moreover, the increasing energy consumption consumed by cloud data centers not only affects the environment by causing global warming, but also increases the cloud user’s cost. Therefore, how to reduce the cloud data center’s energy consumption has become a common concern for government, business and academia in recent years. According to the research in [5], servers operate only at 10%–50% of their capacity most of the time in a data center, and these servers with low utilization cause a great waste of electricity. Therefore, it is feasible to reduce the energy consumption by improving hosts’ resource utilization in cloud data centers.

**Figure 1:**
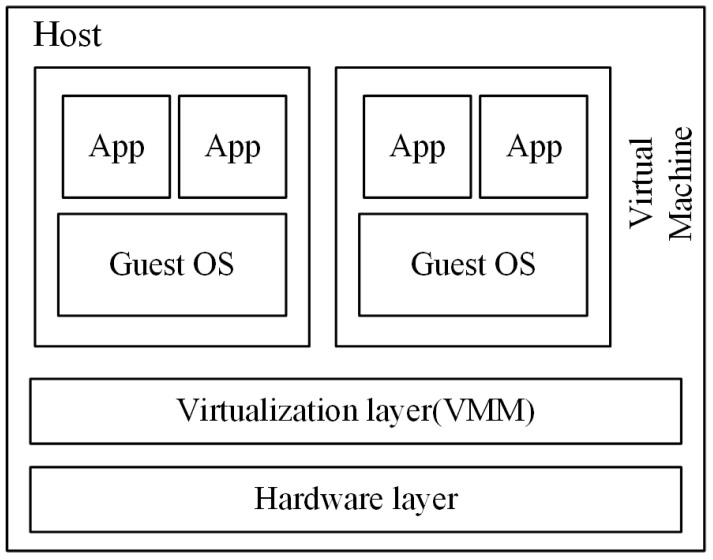
VM abstraction.

Virtualization is the core technology for cloud computing, since it provides a standard view of difference kinds of system resources in cloud data centers. The physical machine (PM), which has been virtualized, can host different kinds of applications by encapsulating them as virtual machines (VM) and keeping them isolated from each other. The process of virtualization is shown in Figure 1; the virtualization layer, which is known as the virtual machine monitor (VMM), is responsible for virtualizing the hardware layer of PMs into virtual resources to be used exclusively by VMs. To function properly, every running VM also requires a certain amount of resources, such as the CPU, memory, storage and bandwidth, but the sum of all required resources should not be beyond the host’s capacity. Moreover, the emerging technology of live migration [6] allows VMs to migrate between different hosts without interrupting the running applications. Therefore, it is convenient to take advantage of the live migration technology to consolidate VMs into a few servers in order to improve the resource utilization of the physical server and to switch off the idle host to reduce the cloud data center’s energy consumption. However, there still exists some problems that should be solved before implementing the VMs’ consolidation:

- In cloud data centers, the system resource requested by cloud users always varies with time. This may result in the resource requirement of the physical server being unstable, especially after consolidating some VMs into the same server. Hence, if the total requested resource is larger than the PM’s capacity, which can be seen as overloaded, and the host may increase response time and reduce throughput, the cloud user may not get their expected QoS, which is defined in terms of the service-level agreement (SLA), and the service provider must get a penalty. Therefore, some policies should be made to avoid the emerging of overloaded hosts and to keep servers relatively stable after VM consolidation.
- The impact of live migration technology on system performance has been discussed in [7]. Generally, the process of live migration consumes some computing resources. Therefore, a large number of VM migrations can consume a large amount of system resources and may cause SLA violations. Therefore, it is necessary to design proper policies to decrease the number of migrations when conducting VM consolidation.

In conclusion, when consolidating VMs into a few servers for energy saving, the cloud provider should take both the dynamic resource requirement and the number of VM migrations into consideration to guarantee the cloud users’ requirement in QoS and to maximize their benefits.

In this paper, we utilize the technology of live migration to implement VM consolidation and improve the resource utilization of physical servers. In addition, we have proposed VM consolidation algorithms, which can satisfy cloud providers’ requirement to decrease energy consumption cost and reduce SLA violations. In brief, the contributions of this paper can be summarized as follows:

- An energy-efficient underloaded host detecting algorithm is proposed in this paper. The algorithm can effectively find proper a underutilized host and migrate out all of the VMs running on the host, then switches the host to sleep mode for power saving. Moreover, it has taken different kinds of PMs into consideration and can be applied to large heterogeneous cloud data centers.
- We propose the remaining utilization-aware (RUA) algorithm to find the new placement for migrating VMs. The RUA algorithm is aware of the number of VMs running on the host and the host’s remaining resources when finding a target host for VMs. Therefore, it is more robust to deal with the variable workload when placing VMs on the physical server.
- These two algorithms have been combined and applied to the cloud data center for reducing both energy consumption and SLA violations. Experimental results have validated the effectiveness of our proposed algorithms.

The remaining part of this paper is organized as follows. Section 2 discusses related work. The system model is presented in Section 3. Our proposed algorithms are introduced in Section 4. Experiments and results are discussed in Section 5. Finally, Section 6 presents the conclusion and future work.

## 2. Related Work

One of the earliest research works about power management in virtualized data centers has been discussed by Nathuji and Schwan in [8]. They explore a way to combine power management mechanisms and policies with the virtualization technologies, which can be actively deployed in a large-scale data center. In their proposed policy, the resource manager is made up of local and global managers. The system simply take the guest OS’s power management strategies as the local manager. Additionally, then, the global manager collects information about the residual resource from local managers for VM placement. However, the specific resource management policy for the global manager has not been discussed in the paper.

Kusic *et al.* [9] have considered the problem of dynamic resource provisioning in cloud data centers as sequential optimization under uncertainty and solved it by using a limited look-ahead control policy. Their objective is to increase the resource provider’s margin by reducing the data center’s energy consumption and SLA violations. In order to improve the QoS, they apply a Kalman filter to estimate the future resource requirement to better conduct resource re-allocations. However, the time complexity of the optimization controller is so high that fifteen nodes consume practically thirty minutes. Hence, it is obviously not suitable for large-scale data centers.

Verma *et al.* [10] regard the problem of power-aware dynamic placement of applications as a bin packing problem, which has variable bin sizes and costs. The technology of live migration is utilized to make VMs migrate from one host to another at each scheduling interval. However, they do not discuss the SLA in the paper. In their recent work [11], the strategy of VM consolidation can be classified as three categories: static consolidation, semi-static consolidation and dynamic consolidation. In static consolidation, VMs are supposed to reside on the host for a long time period. In semi-static consolidation, live migration is used for VM migration in a daily or weekly form, and the dynamic consolidation means that the VM migration would occur at every moment. However, the concrete process of dynamic consolidation is not presented in their paper. Instead, we take advantage of the dynamic consolidation for fine-grained energy optimization in this paper.

Forsman *et al.* [12] propose two algorithms, which can cooperate with each other for conducting automated live migration of multiple virtual machines. In the push strategy, overloaded PMs try to offload a part of their workload to less loaded PMs. Additionally, when the pull strategy is applied, underutilized PMs actively request a great deal of workload from relatively more heavily-loaded PMs. In the proposed algorithms, when and which VMs should be migrated just depend on three factors, that is the PM’s state after migration, the migration cost and the system’s expected workload distribution. The paper focuses on the method to efficiently re-distribute the system’s workload and quickly achieve load balance in the system. However, how to make cloud providers conform to the SLA is not discussed in the paper.

Song *et al.* [13] take advantage of the virtualization technology to dynamically allocate available resources in the cloud data center according to the applications’ requirements and optimize the number of active servers for supporting green computing. They model the process of resource allocation as the relaxed on-line bin packing problem and propose the variable item size bin packing (VISBP) algorithm, which features carefully implemented classifications of VMs and PMs. The algorithm can be tolerant of moderate size variation, as long as the classification rules are kept. Experimental results present that the VISBP has better performance in hot spots’ migration and load balance when compared to the existing algorithm. However, the assumption in the paper that all PMs are homogeneous with unit capacity may be the cardinal limitation of its application.

Huang *et al.* [14] design a sub-optimal dynamic SLA-aware resource allocation strategy for achieving green computing. For improving system performance, the scheduling policy is firstly based on a prediction mechanism of support vector regressions (SVR) to estimate the amount of resource utilization requirements according to the SLA specification. Then, a genetic algorithm-based resource re-allocation mechanism is applied to determine the number of VMs requested by the application services. The proposed scheme can easily satisfy the promised QoS and maximize cloud provider’s profit. Unfortunately, the GA algorithm cannot avoid plunging into the local optimal solution, and its convergence speed is also worthy of discussion.

Islam *et al.* [15] develop novel resource measurement and allocation strategies based on the prediction mechanism for reducing delay in hardware resource allocation. They have applied a neural network and linear regression to predict resource requirement for resource measurement and provisioning strategies, and the experiment results have validated its effectiveness. Therefore, we also apply a prediction-based mechanism to predict the host’s resource utilization status in this paper.

Kinger *et al.* [16] take the physical machine’s working temperature as a criterion to determine whether the virtual machine should be moved and propose a new proactive prediction-based scheduling algorithm. The algorithm takes the current and maximum threshold temperature of the physical machine into consideration before making scheduling decisions and schedules virtual machines among PMs with the help of a temperature predictor to make sure that the threshold temperature of the PM is never reached. Their experiment results show that the proposed algorithm can truly reduce energy consumption and improve the hosts’ resource utilization in the cloud environment. Therefore, the precision of the temperature predictor has a great influence on the system performance.

Beloglazov *et al.* [17] split the problem of dynamic virtual machine consolidation into four parts: (1) determining when a host is considered as being overloaded; (2) finding the underloaded host in the cloud data center; (3) selecting virtual machines that should be migrated from an overloaded and underloaded host; and (4) finding a new placement for those virtual machines. They utilize median absolute deviation (MAD), interquartile range (IR), local regression (LR) and robust local regression (RLR) methods to find the overloaded host and propose the minimum migration time (MMT), random selection (RS) and maximum correlation (MC) policies to choose virtual machines from the overloaded host. For finding the underloaded host, they use a simple method (SM), which aims to select the physical server with the lowest resource utilization as the underloaded host. The power-aware best fit decreasing (PABFD) algorithm is proposed for VM placement. The analysis of the results shows that the combination of MMT for VM selection and LR for finding overloaded hosts has better performance in energy consumption, SLA violation and the number of VM migrations when compared to other combinations.

In this paper, the LR prediction mechanism is applied to detect overloaded hosts, and the MMT policy is used to select VMs from overloaded physical servers. Moreover, the power-aware (PA) algorithm and the remaining utilization-aware (RUA) algorithm are proposed in this paper for underloaded host detection and VM placement, respectively. Since PA and RUA have taken the number of running VMs and the variation of workload into consideration, LR/MMT/PA/RUA can reduce both the number of VM migrations and SLA violations dramatically in comparison to LR/MMT/SM/PABFD.

## 3. System Model

**Figure 2:**
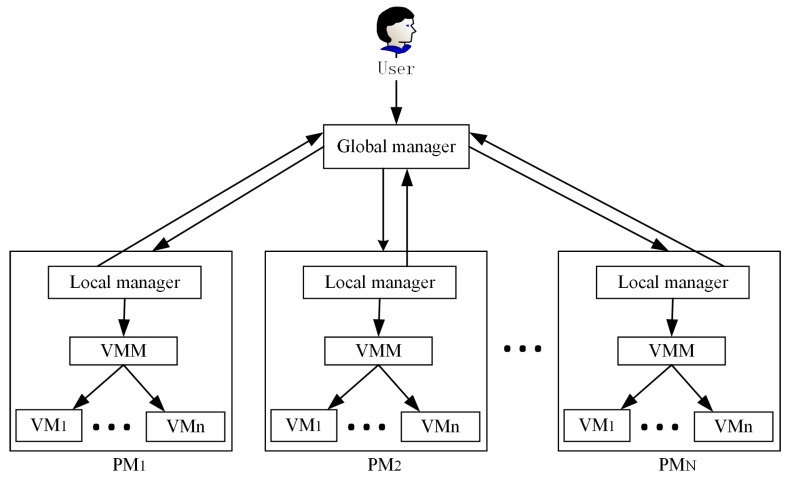
The system model.

In this paper, the definition of the physical node’s resources is described by the CPU, which is denoted by millions of instructions per second (MIPS), random access memory (RAM) and network bandwidth. Besides, network-attached storage (NAS) is applied to represent the PM’s local disks. VMs request these physical resources from the host in response to the user request. As presented in [9] and [18], the PM’s power consumption is nearly proportional to its CPU utilization, so the power consumption can be described by Equation (1):

(1) $$P\left(\mu \right)=0.7\cdot P_{max}+0.3\cdot P_{max}\cdot \mu$$

where *P_max_* denotes the PM’s power consumption when it is in full load. *μ* represents the PM’s CPU utilization, which changes over time. Therefore, we mainly consider the host’s CPU resource to implement VM consolidation for investigating the relationship between cloud data center’s energy consumption and SLA violations in this paper. We use the system model presented in [17], as shown in Figure 2. The software structure of the system is made up of a global and a local manager. There exist N heterogeneous physical servers in a large-scale data center. When users submit tasks to the global manager residing on the master node, then the global manager that collects the local manager’s report about the state of the PM’s resource utilization can make a decision in VM placement or start the process of resource reallocation. The virtual machine monitor (VMM) is utilized to migrate VMs in or out of hosts.

## 4. The Proposed Algorithms

As presented in [17], the procedure of VMs consolidation contains four parts: firstly, finding overloaded hosts in the cloud data center; secondly, selecting some VMs to be migrated from overloaded hosts; thirdly, finding underloaded hosts and selecting all virtual machines running on them for migration; lastly, these selected VMs are placed on appropriate hosts. Since the VM consolidation policy aims to improve resource utilization and reduce the number of active physical servers, the minimum number of running hosts can be obtained by solving the following linear programming equation:

(2) $$\sum_{i=1}^{n}R_{vm_{i}}^{cpu}\leq \sum_{j=1}^{m}x_{j}\cdot C_{j}$$

(3) $$P_{total}=\sum_{i=1}^{m}x_{i}\cdot P_{i}$$

(4) $$Objective:minimize\{P_{total}\}$$

In the above equation, $R_{vm_{i}}^{cpu}$ denotes the amount of CPU resource requested by the *i*-th VM. $C_{j}$ represents the capacity of the *j*-th kind of physical server, and *x_j_* represents the number of the *j*-th kind of physical server used. *P_i_* denotes the power consumption of the *j*-th kind of host, which can be calculated by Equation (1). We can get the minimum number of each kind of active host by minimizing the *P_total_*. However, the minimum number of hosts that can be obtained by the above equation may not be applicable to the data center when the users’ resource requirement varies with time. In order to strictly adhere to SLA, some VMs have to be migrated from overloaded hosts, and hosts that are in sleep mode need to be activated for VM placement once the workload increases. That can generate the extra number of VM migrations and frequently switch the host on or off. Therefore, a novel approach is presented to reduce energy consumption and the number of migrations in this section.

### 4.1. Algorithm for Underloaded Host Detecting

Before finding underloaded hosts for VM consolidation in a cloud data center, overloaded hosts should be identified by the LR method, and some VMs should be selected for migrating from overloaded hosts according to the MMT policy, which has been proposed in [17]. When overloaded hosts have been dealt with, the underload detection algorithm starts to work. We propose the power-aware (PA) algorithm to detect the underloaded host by comparing the ratio of the host’s power consumption to the number of VMs running on the host among active physical servers in the cloud data center and then try to place all of the VMs from the underloaded host on the other active hosts. If this could be done without causing target hosts to be overloaded, then we switch the underloaded host off once all VMs have been placed on the target hosts. Otherwise, VM migration is canceled, and underloaded hosts keep running. PA is executed repeatedly to reduce the number of active physical servers for energy saving in the cloud data center. Equation (5) is proposed to calculate the power-efficient value (*PE*) of the active host, which can be used to choose the underloaded host.

(5) $$PE_{j}=\frac{P_{j}}{M_{j}}$$

In the above equation, *P_j_* represents the power consumption of the *j*-th host in the cloud data center, and *M_j_* represents the number of VMs running on the *j*-th host.

In order to select the proper underloaded host and facilitate the VM consolidation, the lower threshold (*α*) of the CPU resource utilization should be set to specify the search scope of PA. Then, if the CPU resource utilization of the host is not larger than the *α* value, then the host can be added to a candidate host set. At last, Equation (5) is applied to calculate the *PE* value of the host selected from the candidate host set. The host that has the maximum *PE* value can be chosen as the underloaded host.

Obviously, since PA has taken the host’s current power consumption and the number of VM migrations into consideration, it has the following advantages when detecting the underloaded host:

- Compared to the SM used in [17], the *PE* value proposed in the paper can evidently exhibit the host’s contribution for energy saving in the process of underloaded host detecting. Particularly, when there exists more than one host with the same least CPU utilization in a heterogeneous cloud data center, PA can efficiently select the host with higher power consumption and a lesser number of VMs as the underloaded host to switch it off. However, the SM is not good at handling the common case.
- PA has considered the number of VMs running on the host as another key indicator. The host with more VMs has a lesser *PE* value, so it has fewer chances to be selected as the underloaded host. Since the process of VM migration also consumes system resources, a large number of migrations may reduce system performance and increase SLA violations. Therefore, PA can satisfy QoS and reduce the number of migrations by switching the appropriate host to sleep mode. In addition, resource utilization of the host running more VMs may fluctuate frequently with time due to the variability of the workload. In other words, this kind of host that can be treated as underutilized at the current time may become overloaded the next time. Therefore, it is wise to exclude the host with more VMs from being selected as the underloaded host. The designed *PE* value is aware of the number of VM migrations and is likely to choose the host that has a lesser number of VMs and more power consumption among the underutilized hosts as the underloaded host. Hence, PA can reduce SLA violations and improve the system performance.

### 4.2. Algorithm for VM Placement

**Figure 3:**
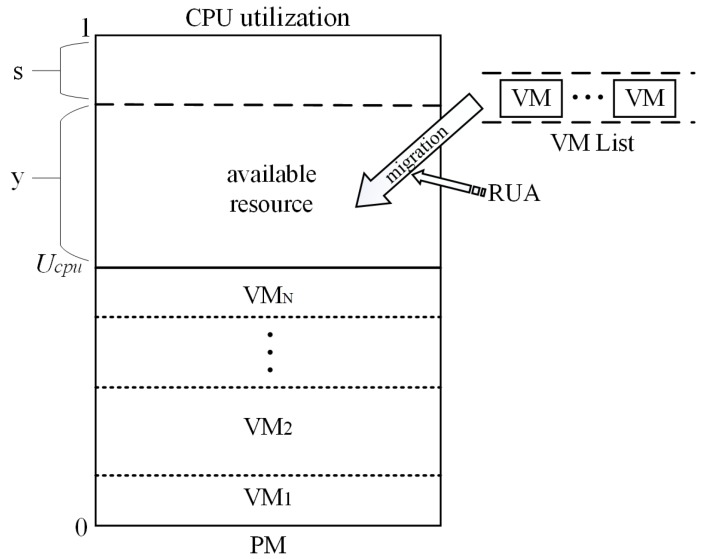
CPU utilization model of the physical machine (PM).

In order to improve the stability of hosts after VM placement and reduce the SLA violations, a remaining utilization-aware (RUA) VM placement algorithm is proposed to place VMs on appropriate physical servers. The CPU resource utilization model of the physical node is introduced firstly in this part for conveniently illustrating the working principle of RUA, as shown in Figure 3.

*U_cpu_* denotes the PM’s current CPU resource utilization; VM_1_,VM_2_...and VM_*N*_ represent that there exist *N* VMs running on the PM at present and each of them consumes much of the CPU resource of the physical server; *y* represents the remaining available CPU resource in the PM; VM Listis used to restore VMs that need to be migrated; *s* is the safety parameter, which prevents the performance of the host from degrading [19]. In order to keep consistent with the safety parameter used in the LR, which has been discussed in [17], the value of *s* is set to be 0.17 in this paper.

RUA can be divided into two steps. First, the candidate host list is created by choosing hosts in the cloud data center. The candidate host is the one who has available CPU resource allocated to the newly-coming VM, and the underloaded host should be excluded from the candidate host list. Then, the process of finding the host for VM placement can be divided into two cases:

Case 1: if a host’s CPU utilization status $\frac{U_{cpu}}{N}>y$, the VM whose CPU utilization request from the host is less than $\frac{U_{cpu}}{N}$ can be placed on the host;

Case 2: if a host’s CPU utilization status $\frac{U_{cpu}}{N}\leq y$, the VM whose CPU utilization request from the host is not less than $\frac{U_{cpu}}{N}$ can be placed on the host;

If some VMs still cannot find a suitable host after traversing the candidate host list, then PABFD [20] is applied to complete VM migration. Furthermore, all of the VM placements are supposed to keep the target host from overloading. The pseudo-code of RUA is presented in Algorithm 1.

RUA aims to improve the PM’s resource utilization by placing VMs on less hosts. Meanwhile, it takes host’s resource utilization status into account and tries to improve average utilization, as well as avoid putting too many VMs on the same host. Hence, RUA can tolerant the variable workload and reduce the number of migrations among hosts. On the other hand, RUA can also prevent placing VMs that have a large resource request on the same host for reducing resource competition among VMs. It can make the probability of server overloading decrease, keep server’s status relatively stable and reduce SLA violations, as well. The complete procedure of VM consolidation is presented in Algorithm 2.
**Algorithm 1:** RUA placement.**1:****Input:** candidate_hostList,vmList **Output:** migrationSched**2:****for each:** vm *in* vmList **do****3:** allocatedHost ← NULL**4:** CPURequired ← vm.getCPUrequired()**5:** **for each:** host *in* hostList **do****6:**  **if** host has enough resource for vm **then****7:**   CPUratio ← CPURequired/host.getTotalCPU()**8:**   **if**
$\frac{U_{cpu}}{N}>y$
**then****9:**    **if** CPUratio < $\frac{U_{cpu}}{N}$
**then****10:**     allocatedHost ← host**11:**    **end if****12:**   **else then****13:**    **if** CPUratio > = $\frac{U_{cpu}}{N}$
**then****14:**     allocatedHost ← host**15:**    **end if****16:**   **end if****17:**  **end for****18:**  **if** allocatedHost ≠ NULL **then****19:**   migrationSched.add(vm,allocatedHost)**20:**  **else then****21:**   migrationSched.add(**PABFD**(vm,allocatedHost))**22:**  **end if****23:** **end for****24:** **return** migrationSched
**Algorithm 2:** Integrated VM consolidation.**1:****Input:** hostList **Output:** scheduleMap**2:****for each:** host *in* hostList **do****3:** **if LR**(host) is overloaded **then****4:**  **while** host is overloaded **do****5:**   VM ← **MMT**(host)**6:**   migrationList.add(VM)**7:**  **end while****8:** **end if****9:****end for****10:**scheduleMap.add(**RUA**(migrationList))**11:**Obtain candidateHostSet from hostList**12:****for each:** host selected from candidateHostSet **do****13:** **if PA**(host) is underloaded **then****14:**  **if RUA**(host.getVmList()) succeeds **then****15:**   scheduleMap.add(**RUA**(host.getVmList()))**16:**   Switch the host to sleep mode**17:**  **end if****18:** **end if****19:****end for****20:****return** scheduleMap


## 5. Experiments and Results

### 5.1. Experimental Setup

Since the system is applied to the IaaS environment, there may be a large number of hosts and VMs running in response to the user’s requests. It is necessary to simulate the kind of application scenario and output the repeatable results when choosing simulation tools. Furthermore, it should be a completely customizable tool.

To evaluate the effectiveness of algorithms proposed in this paper, we choose CloudSim as the simulation platform. It is an extensible simulation toolkit that enables modeling and simulating of the cloud computing systems, provides the applications’ environments and supports both system and behavior modeling of cloud systems, such as data centers, virtual machines and resource provisioning policies [21]. We have used 800 heterogeneous physical servers and five types of virtual machines in CloudSim to simulate the cloud data center. Half of these physical nodes are HP ProLiant ML110 G4 servers, and the other nodes are HP ProLiant ML110 G5 servers. In addition, these virtual machine types used in the paper are similar to Amazon EC2 instances. That is, high-CPU medium instance (2500 MIPS, 0.85 GB), extra large instance (2000 MIPS, 3.75 GB), small instance (1000 MIPS, 1.7 GB) and micro instance (500 MIPS, 613 MB). When the resource requirement of VMs varies, the PA and RUA algorithms try to consolidate different VMs into fewer hosts for energy saving.

### 5.2. Workload Data

In order to validate the PA and RUA algorithms, the workload data, which are used for simulation, are extracted from the CoMonproject, a monitoring infrastructure for PlanetLab [22]. These data are about CPU utilization, which is measured from thousands of virtual machines running on different servers around the world. The characteristics of workload data, which have been presented in [23], are shown in Table 1. From Table 1, we can see that the mean value of workload traces is quite small, most everyday values of the traces are almost zero, and these traces vary from 0 to 99. That means we should consider the situation of overloaded hosts caused by the variable workload when applying the VM consolidation scheme to workload traces.

### 5.3. Performance Metrics

Quantifying the quality of the cloud service is indispensable for both the cloud service provider and the consumer to reach the desirable SLA, which can be defined as the maximum response time or minimum throughput, before deploying the system. Furthermore, it can be used to judge whether the cloud provider deserves the payment or punishment. As described in [17], this quantification can be defined as *SLAV*, which is a workload independent metric shown in Equation (6):

(6) SLAV=SLATAH×PDM

where *SLATAH* denotes the average ratio of the period when the host experiences CPU utilization of 100%, as is shown in Equation (7):

(7) $$SLATAH=\frac{1}{N}\sum_{i=1}^{N}\frac{T_{si}}{T_{ai}}$$

where *N* represents the number of hosts, *T_si_* is the time span for which the host’s utilization reaches 100% and *T_ai_* represents the time in which the host i is active. In addition, *PDM* denotes the performance degradation due to VM migrations, which can be calculated by Equation (8):

(8) $$PDM=\frac{1}{M}\sum_{i=1}^{M}\frac{C_{di}}{C_{ri}}$$

where *M* represents the number of virtual machines and *C_ri_* means the total CPU utilization requested by the *i*-th VM. *C_di_* is the performance degradation caused by VM migrations. According to the research about VM migration presented in [7], we set *C_di_* to be 10% of the total CPU utilization in MIPS during the migration of the *i*-th VM in the experiment.

Since the object of VM consolidations in cloud data centers is to save energy and improve QoS, so we use minimizing energy consumption and reducing SLA violations to measure, another combined metric presented in [17] is used to measure the system performance, which is defined in Equation (9):

(9) ESV=E×SLAV

### 5.4. Results and Analysis

**Figure 4:**
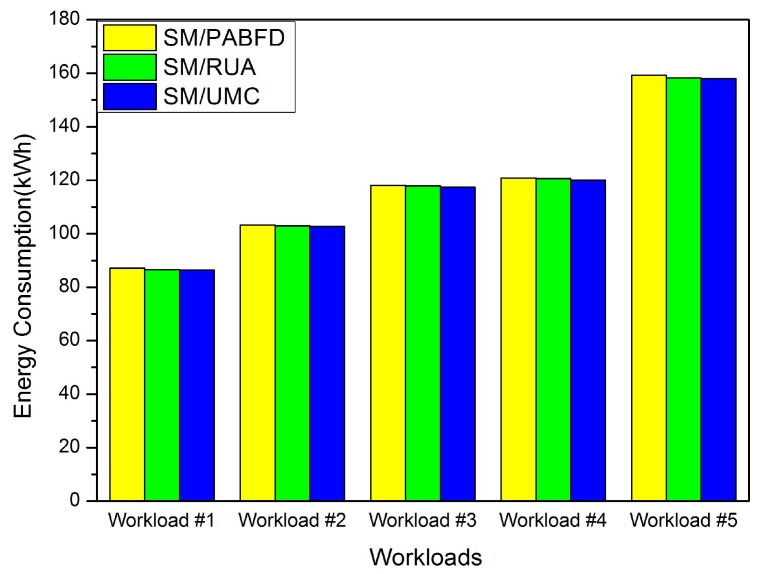
Energy consumption.

**Figure 5:**
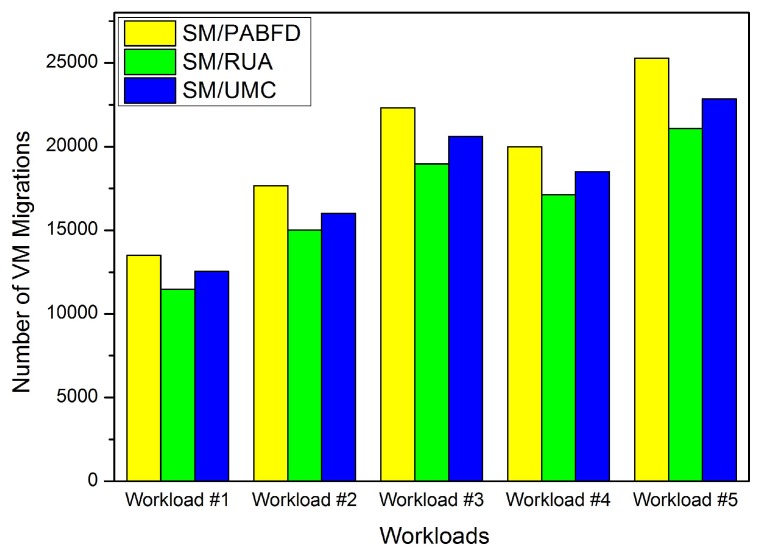
The number of VM migrations.

**Figure 6:**
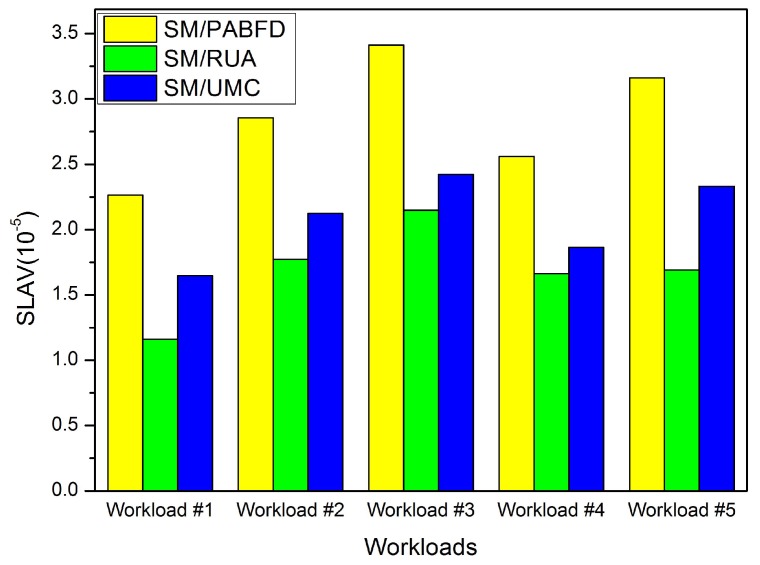
SLAV.

**Figure 7:**
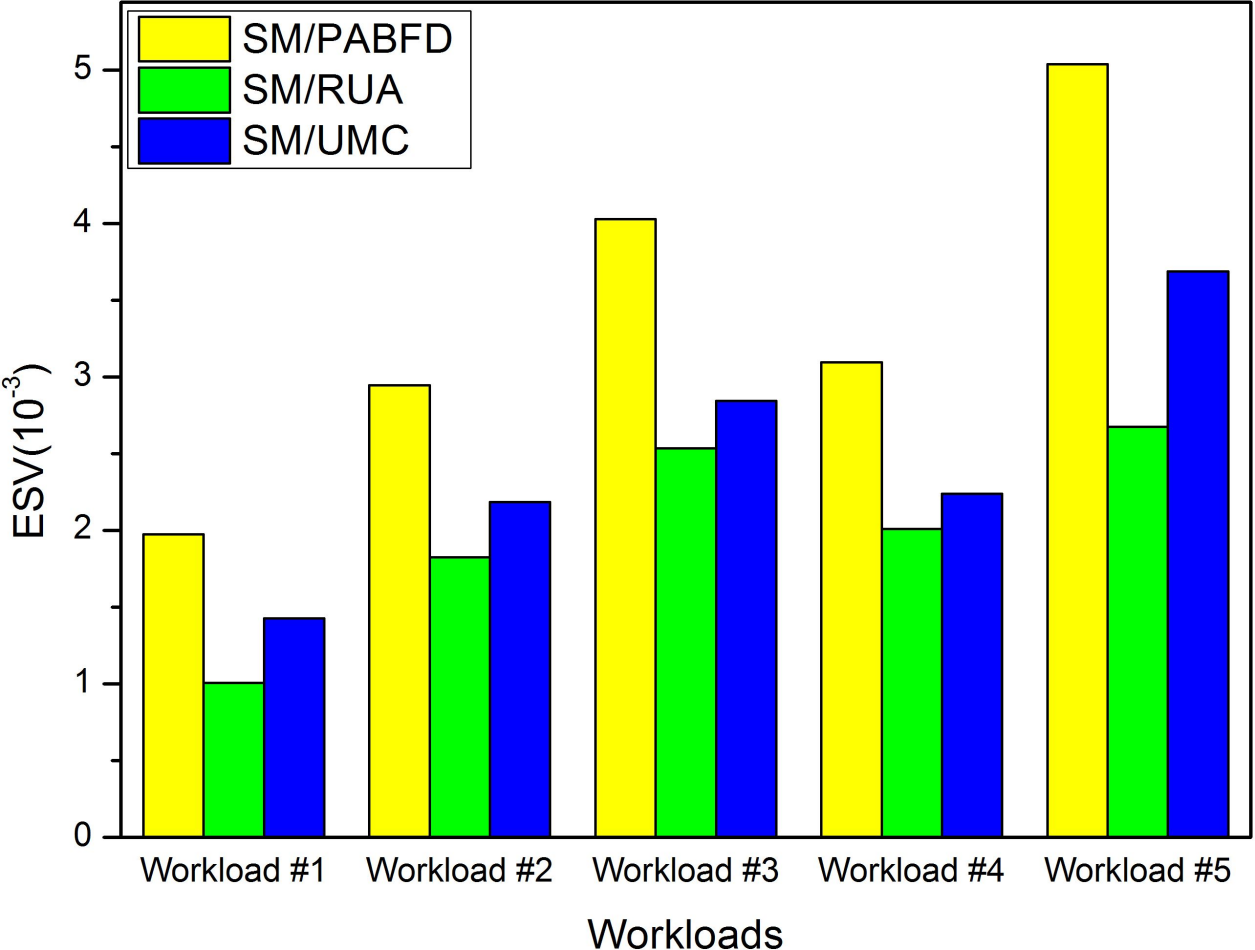
ESV.

In this section, we conduct some experiments to evaluate the effectiveness of the PA and RUA algorithms in the cloud environment. Firstly, we compare RUA to PABFD and the utilization and minimum correlation (UMC) algorithm, which is presented in [23] based on the idea proposed by Verma *et al*. [11] that the higher the correlation between applications running on an oversubscribed server for completing the same kind of resource, the higher the probability of the server overloading. We choose five days’ workload from PlanetLab as five different kinds of workloads to test the three algorithms (PABFD, RUA and UMC), and each kind of algorithm is combined with SM to accomplish VM consolidation. Experiment results are shown in Figure 4, Figure 5, Figure 6 and Figure 7.

Figure 4 shows the energy consumption of the three algorithms when different workloads are applied in the cloud data center. The energy consumption of RUA and UMC is almost the same, but less than PABFD. Hence, RUA can the reduce energy consumption and total cost for the cloud provider by finding the proper host for VM placement.

Figure 5 shows the number of VM migrations scheduled by three algorithms during the entire simulation. We see that the number of live migrations scheduled by RUA is obviously less than the other two algorithms. This is because RUA is more robust to handling a variable workload and can efficiently reduce the amount of overloaded hosts by adjusting the number of VMs running on the physical servers. When VMs’ resource requirements change, UMC and PABFD are more subtle than RUA to reschedule VMs to prevent hosts from overloading. Therefore, the number of live migrations scheduled by RUA are reduced obviously.

Figure 6 depicts SLA violations using PABFD, RUA and UMC, respectively. Since RUA schedules a lesser number of VM migrations, so it is more robust to dealing with a variable workload to prevent hosts from overloading, it has less SLA violations than the other two algorithms. That means that RUA has better performance with respect to SLA.

Figure 7 shows the ESVmetric values of the three algorithms in different workload cases. We see that RUA has the least ESV value compared to PABFD and UMC. This is because RUA has less SLA violations and consumes less energy than UMC and PABFD. This figure also denotes that RUA is much better than the other two algorithms.

Then, in order to test the validity of the RUA and PA algorithms, we conduct another set of experiments in the paper, and the results are shown in Table 2. The combinational algorithm of SM and RUA (SM/RUA) has been applied to allocate VMs among physical servers in the cloud data center; it works well and reduces energy consumption, the number of VM migrations and the ESV value simultaneously when compared to SM/PABFD. PA aims to select the physical server that consumes more power and has a lesser number of running VMs as the underloaded host; thus, it is necessary to set a proper *α* value to prevent the host that is close to the overloaded status from being selected as the underloaded host. We set the *α* value to decrease from 0.7 to 0.3, because the small *α* value is disadvantageous to reduce the energy consumption in the cloud data center. Therefore, PA with different *α* values is combined with RUA (PA/RUA-*α*) to discuss the relationship between energy consumption and SLA violation in this part.

**Figure 8:**
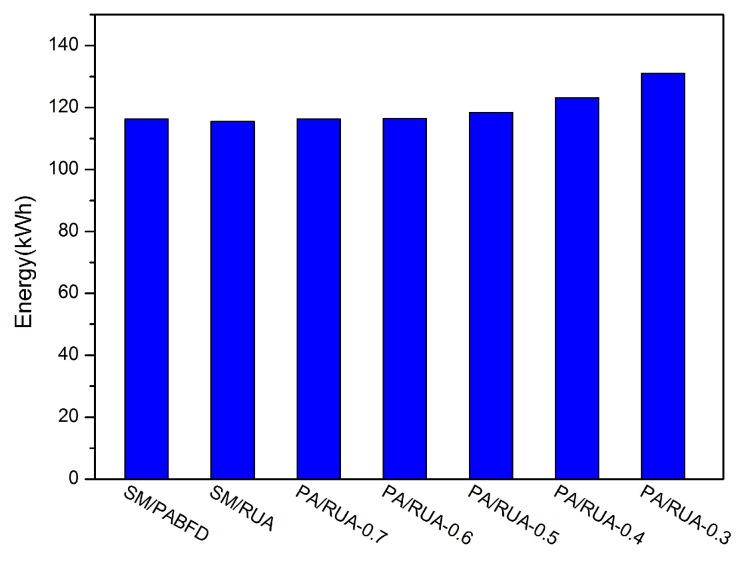
Energy consumption.

**Figure 9:**
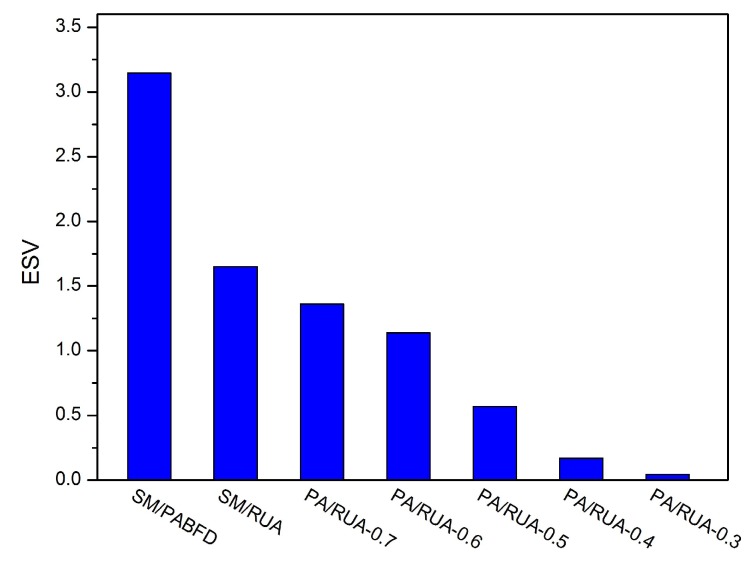
ESV.

**Figure 10:**
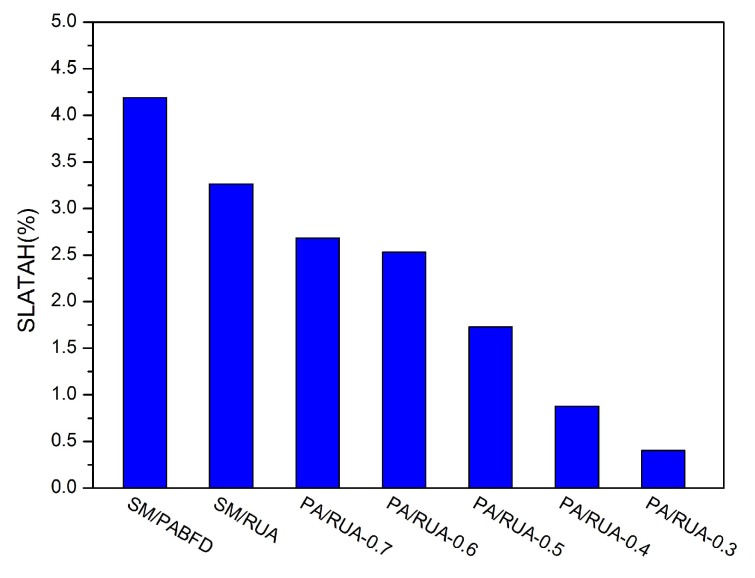
SLATAH.

**Figure 11:**
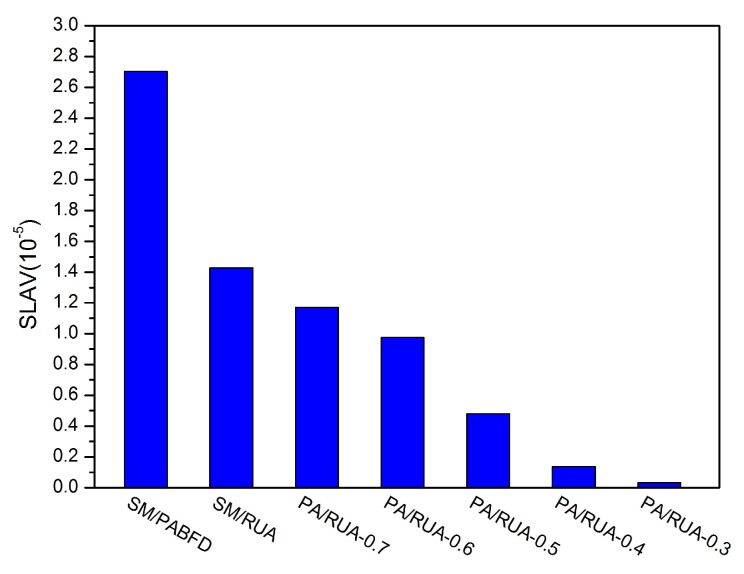
SLAV.

**Figure 12:**
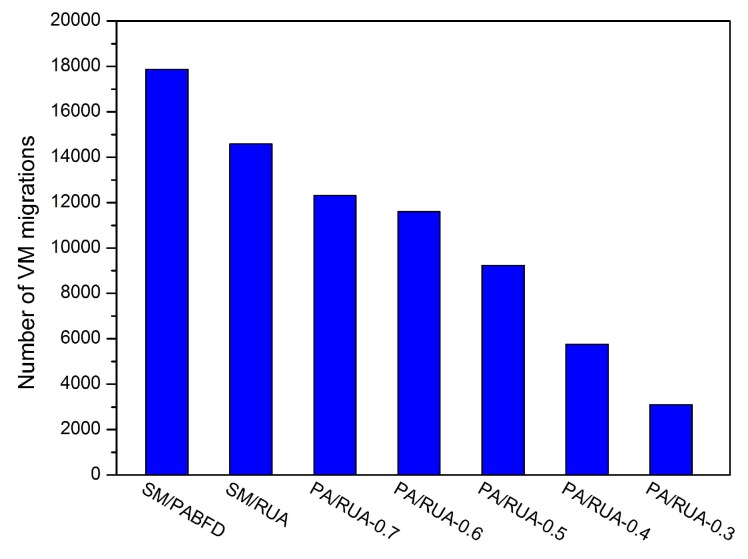
The number of VM migrations.

Figure 8, Figure 9, Figure 10, Figure 11 and Figure 12 present the variation tendency of energy consumption, the number of VM migrations, SLAV, SLATAHand ESV with different *α* values, respectively. We can see that when the *α* value varies from 0.7 to 0.3, the energy consumption in the cloud data center changes very little. However, the number of VM migrations, SLA violation, SLAV time per active host and ESV reduce significantly. This is because when the *α* value decreases, the search scope of PA descends gradually, as well. That can help PA to choose the host that has a fair amount of remaining CPU resource and fewer running VMs as the underloaded host. Hence, the number of VM migrations drops to a large extent, which can reserve a fair amount of CPU resource consumed by the procedure of live migration and results in SLAV decreasing dramatically, as well. Figure 9 shows that PA/RUA is much better than SM/PABFD with respect to energy consumption and SLA violation because the ESV value declines greatly when the *α* value varies. Moreover, Figure 8 and Figure 11 depict that when the energy consumption in the cloud data center increase slightly, the SLA violation reduces quickly. The cloud service provider should make a trade-off between reducing energy consumption and ensuring the user’s QoS.

**Figure 13:**
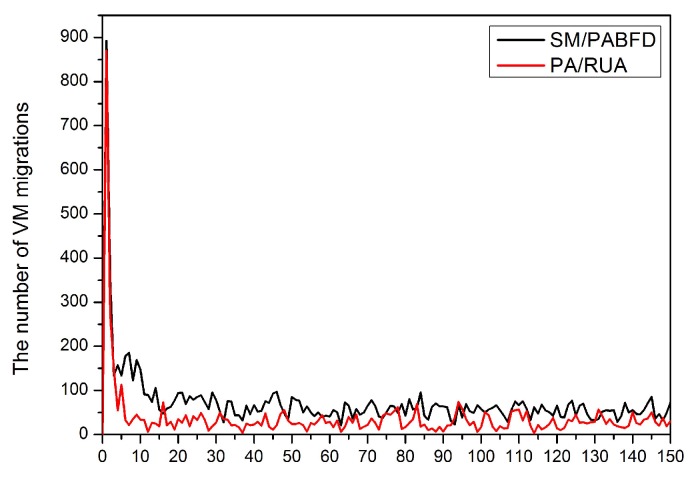
The number of VM migrations varies with time.

Figure 13 shows that the number of VM migrations varies in every scheduling interval. At the initial scheduling phase, both SM/PABFD and PA/RUA have a large number of migrations. That means the optimization algorithm has an effect, and VMs are consolidated into hosts. In the remaining scheduling time, the number of VM migrations scheduled by both SM/PABFD and PA/RUA reduces greatly because there is scarcely any scope for switching active PMs to sleep mode, and the scheduling algorithms focus on dealing with the variation of hosts’ resource utilization caused by the variable workload in those consolidation intervals. Moreover, we can see that PA/RUA schedules a lesser number of live migrations than SM/PABFD, which denotes that PA/RUA can make physical servers more robust to handling the variable resource requirement of VMs compared to SM/PABFD.

## 6. Conclusions

Consolidating VMs to a few hosts and switching idle physical nodes off are the direct methods for cloud providers to decrease energy consumption and reduce cloud user’s operation cost. However, massive oversimplified consolidations can result in violations of SLA and punishment of cloud providers. In this paper, we discuss the approach to reduce energy consumption and SLAV and propose PA for detecting underloaded hosts, as well as RUA for VM placement. The simulation results have shown that PA/RUA can indeed reduce SLA violations and improve the system performance significantly while saving energy.

In the future, we intend to evaluate PA/RUA in a real cloud infrastructure and to help the cloud providers make better management policies. Furthermore, we plan to discuss the effect of other system resource on both energy consumption and SLA violations and take the virtual machine’s communication overhead into consideration.

## Figures and Tables

**Table 1:** Characteristics of the workload (CPU utilization).

Data	The number of VMs	Mean (%)	SD (%)	Most (ratio)	Range (%)
03/03/2011	1052	12.31	17.09	2 (16.6%)	99
06/03/2011	898	11.44	16.83	2 (18.1%)	99
09/03/2011	1061	10.70	15.57	0 (18.2%)	99
22/03/2011	1516	9.26	12.78	0 (18.9%)	99
25/03/2011	1078	10.56	14.14	0 (15.0%)	99
03/04/2011	1463	12.39	16.55	0 (14.2%)	99
09/04/2011	1358	11.12	15.09	0 (17.0%)	99
11/04/2011	1233	11.56	15.07	0 (15.2%)	99
12/04/2011	1054	11.54	15.15	0 (15.4%)	99
20/04/2011	1033	10.43	15.21	0 (17.6%)	99

**Table 2:** Experiment results. SM, simple method; PABFD, power-aware best fit decreasing; RUA, remaining utilization-aware.

Algorithm	Energy (kWh)	VM Mig	SLAV (10^−5^)	SLATAH(%)	ESV (10^−3^)
SM/PABFD	116.35	17,879	2.7036	4.192	3.1456
SM/RUA	115.58	14,584	1.4277	3.263	1.6501
PA/RUA-0.7	116.31	12,319	1.1709	2.685	1.3619
PA/RUA-0.6	116.52	11,608	0.9760	2.532	1.1372
PA/RUA-0.5	118.47	9239	0.4811	1.729	0.5699
PA/RUA-0.4	123.19	5755	0.1373	0.879	0.1692
PA/RUA-0.3	131.06	3102	0.0334	0.404	0.0438
